# Association of blood vitamin A with osteoarthritis: a nationally representative cross-sectional study

**DOI:** 10.3389/fnut.2024.1459332

**Published:** 2024-11-05

**Authors:** Ao Wu, Ning-ning Wu, Peng-hui Xu, Yao Jin, Zhi-kai Yang, Jia-wen Teng

**Affiliations:** ^1^The First Clinical College of Shandong University of Traditional Chinese Medicine, Jinan, China; ^2^Affiliated Hospital of Shandong Traditional Chinese Medicine University, Jinan, China; ^3^Shengli Oilfield Central Hospital, Dongying, China

**Keywords:** vitamin A, osteoarthritis, NHANES, nutritional assessments, public health

## Abstract

**Objectives:**

Vitamin A plays an important role in health, especially regarding its impact on bone tissue. Vitamin A can lead to bone damage and deformity, thus becoming an important causative factor in osteoarthritis. In this study, we aimed to evaluate the association of serum vitamin A with osteoarthritis.

**Methods:**

We included participants who self-reported whether they had OA in NHANES 2001–2006 and NHANES 2017–2018 to explore the association and dose–response relationship between vitamin A concentration and risk of osteoarthritis through weighted multivariate logistic models and restricted cubic splines. Sensitivity and stratification analyses were also used to assess the robustness of the results.

**Results:**

A total of 18,034 participants were included in this study, and a linear association between serum vitamin A concentration and osteoarthritis risk was observed. The OR of osteoarthritis was 1.22 (95% CI: 0.98, 1.52), 1.40 (95% CI: 1.05,1.85), and 1.47 (95% CI: 1.14, 1.91) for participants in the second, third, and fourth quartiles, respectively, compared with the lowest vitamin A reference group. Similar results were obtained when sensitivity and stratification analyses were performed.

**Conclusion:**

Serum vitamin A is positively associated with osteoarthritis risk. Within a certain range of vitamin A concentrations, vitamin A is a protective factor against osteoarthritis, beyond which it becomes a causative factor for osteoarthritis.

## 1 Introduction

Osteoarthritis (OA) is a disease that is prevalent among older adults and a major cause of decreased physical functioning (1). Approximately 527.8 million people were affected globally in 2019 (2), and it has affected 32.5 million adults in the United States (3). Osteoarthritis has seriously affected people’s quality of life, causing a huge social and personal burden (4). Osteoarthritis is influenced by age, gender, race, joint structure, obesity, and vitamins (5).

Vitamin A, which is found both in foods of animal origin and in plants, is a substance with retinol bioactivity (6). Vitamin A plays a crucial role in controlling cell growth and differentiation in numerous cells, and it is particularly important for embryonic development, notably in the process of bone formation (7, 8). However, vitamin A also has harmful effects on bone, and many studies have shown that increased serum levels of vitamin A lead to increased osteoclast formation, which in turn reduces cortical bone mass leading to increased bone fragility (9–11).

Osteoarthritis involves lesions of the cartilage, subchondral bone, meniscus, and surrounding soft tissues. Articular cartilage provides a smooth surface for movement, while the subchondral bone offers stability to the articular cartilage, and the subchondral bone is critical to the development of osteoarthritis. In the usual case, there is a delicate balance between the breakdown of bone by osteoclasts and the formation of bone by osteoblasts, which is essential for maintaining bone health (12). When the number of subchondral osteoclasts increases, the rate of bone resorption is significantly accelerated, which disrupts the balance of bone remodeling and the homeostasis of the subchondral microenvironment (13), further affects angiogenesis and innervation, and accelerates articular cartilage damage (14, 15). On the other hand, enhanced bone resorption leads to irregularities in the articular cartilage, leading to the formation of bone cysts, which in turn cause joint discomfort and pain. Meanwhile, vitamin A causes an increase in osteoclasts, and studies by previous scholars have demonstrated that vitamin A is associated with osteoporosis and an increased risk of fracture (6, 10, 11). A study showed elevated levels of vitamin A metabolites in the synovial fluid of patients with OA (16). A study by Kulaklı et al. (17) showed that isotretinoin (a vitamin A derivative) is associated with the formation of osteoarthritis. Previous studies by scholars had limited sample sizes and lacked large-scale population validation; therefore, in this study, we used a large-scale population sample to assess the association between vitamin A concentrations and osteoarthritis risk. This study may help clinicians assess patients’ vitamin A levels at an early stage, leading to early intervention and monitoring of high-risk populations, and may also provide new perspectives for research in the fields of nutrition and public health.

## 2 Materials and methods

### 2.1 Research participants

The National Health and Nutrition Examination Survey (NHANES) is a nationwide survey administered by the National Center for Health Statistics (NCHS) to furnish national health data. The NHANES data utilized in this research can be openly accessed through the link: https://www.cdc.gov/nchs/nhanes/ (accessed on February 14, 2024). An ethical protocol approved by the NCHS Research Ethics Review Board was implemented and all recruited participants signed an informed consent form.

**Figure 1:**
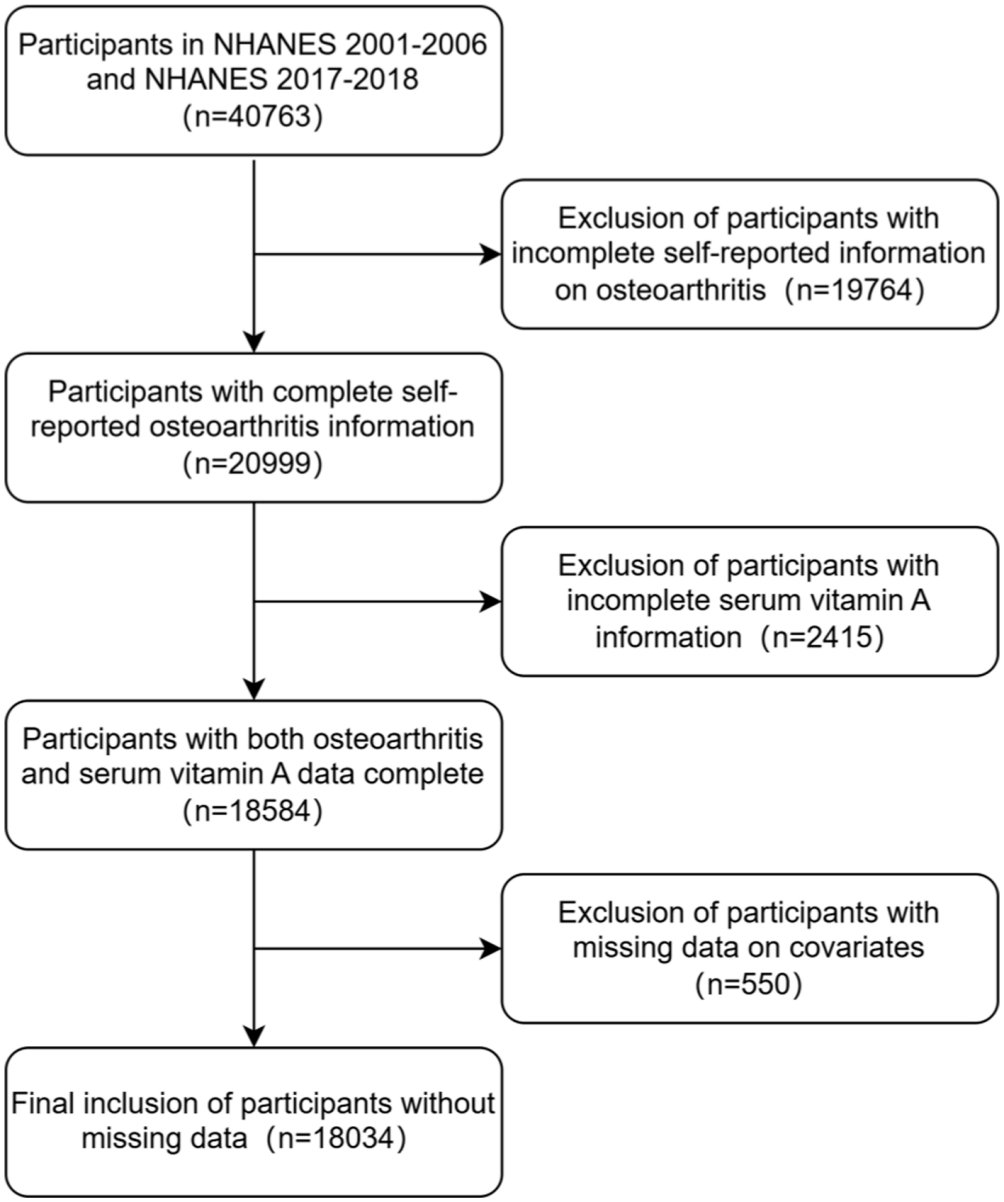
Participant screening flowchart.

In this study, we examined data from four cycles of NHANES 2001–2006 and NHANES 2017–2018. A total of 40,763 participants were enrolled in these four cycles of NHANES surveys. We excluded patients with missing data on serum vitamin A or osteoarthritis (*n* = 22,179) and further excluded participants with missing data on covariates (*n* = 550), finally, a total of 18,034 participants were included. The flow chart of the study is shown in Figure 1.

### 2.2 Measurement of vitamin A

Participant serum samples for vitamin A measurement were collected at the Mobile Examination Center (MEC). During the NHANES 2001–2002 and NHANES 2003–2004 cycles, serum specimens were processed, stored in vials at −20°C, and shipped to the Centers for Disease Control and Prevention (CDC). Serum specimens from NHANES 2001–2002 participants were sent to CDC for analysis of vitamin A using HPLC methods, and serum specimens from NHANES 2003–2004 participants were sent to Craft Technologies, Inc.(CTI) for analysis of vitamin A using similar HPLC methods. During the NHANES 2005–2006 and NHANES 2017–2018 cycles, serum specimens were processed, stored in vials at −30°C, and analyzed at The Division of Environmental Laboratories at CDC in Atlanta, Ga using HPLC methods for vitamin A Analysis.

### 2.3 Assessment of osteoarthritis

Self-reported OA diagnoses were determined by the following questions: “Has a doctor or other health professional ever told you that you had arthritis?,” if the participant answered “Yes,” they were then asked, “Which type of arthritis was it?.” Participants were categorized as self-reported OA if they answered “Osteoarthritis” during the NHANES 2001–2002, NHANES 2003–2004, and NHANES 2005–2006 cycles. In the NHANES 2017–2018 cycle, participants were also categorized as self-reported OA if they answered “Osteoarthritis or degenerative arthritis.”Previous scholars have shown that self-reported OA has an 85% concordance rate with clinically diagnosed OA (18), suggesting that self-reported OA results are highly credible.

### 2.4 Covariates

Based on prior academic studies, various possible variables that could potentially influence the results were taken into consideration. Demographic information, including age, gender, and race, was collected, categorizing age according to a cutoff of 60 years (divided into ≥60 and <60) and race as Mexican American, Other Hispanic, Non-Hispanic White, Non-Hispanic Black, and Other Race. According to the World Health Organization’s definition of BMI thresholds, BMI is categorized as underweight (BMI <18.5), normal weight (18.5–25), overweight (25–30), and obesity (BMI >30). Based on the response to the query “Have you ever smoked a minimum of 100 cigarettes in your lifetime?,” smoking is classified as “Yes (Smoked ≥100 cigarettes in life)” and “No (Smoked <100 cigarettes in life).” Hypertension was defined as the response to the question, “Have you ever been told by a doctor or other health professional that you had hypertension, also called high blood pressure?,” divided into two groups (Yes or No). The responses to the question “Other than during pregnancy, have you ever been told by a doctor or health professional that you have diabetes or sugar diabetes?” were categorized into two groups (Yes or No). Heart failure, coronary heart disease, and stroke were each defined as answers to the following questions: “Has a doctor or other health professional ever told you that you had congestive heart failure?” “Has a doctor or other health professional ever told you that you had coronary heart disease?” “Has a doctor or other health professional ever told you that you had a stroke?,” were categorized into two groups (Yes or No).

### 2.5 Statistical analysis

The analysis of the statistical data was carried out using R software (version 4.4.0), and to ensure that the results were nationally representative, the data analysis took into account sampling weights. Serum vitamin A concentrations were divided into four groups according to quartiles (≤45.10 μg/dl; 45.10–55.40 μg/dl; 55.40–67.40 μg/dl; and ≥67.40 μg/dl) and analyzed for baseline characteristics. Categorical variables were tested using chi-square tests and proportions were used to describe categorical variables. Independent associations between vitamin A and osteoarthritis were assessed using multifactorial logistic regression analysis with different covariate correction models. Vitamin A concentration was used as a continuous variable, and restricted cubic spline plots were drawn to assess the nonlinear relationship between vitamin A concentration and the risk of osteoarthritis, a process that adjusted for confounders. Multivariate logistic regression analysis was utilized to conduct subgroup and interaction analyses. Furthermore, sensitivity analyses were conducted to verify the reliability of the findings as outlined below: (1) to avoid the effect of extreme vitamin A concentrations, data other than mean ± standard deviation × 3 were excluded; (2) because pregnancy affects serum vitamin A concentrations, participants who were pregnant were excluded; (3) Participants with BMI <15 and BMI ≥40 were excluded, taking into account the possible influence of fragile and excessively obese patients on the results. The independent association between vitamin A and osteoarthritis was then assessed using weighted multifactor logistic regression analysis with different covariate-corrected models using the method of taking quartiles described above. To further validate the accuracy of the results, we performed an unweighted multifactor logistic regression analysis to assess the association between vitamin A and osteoarthritis. A *p*-value of less than 0.05 was considered statistically significant.

## 3 Results

### 3.1 Baseline characteristics

A table of baseline characteristics stratified by serum vitamin A concentration levels is shown in Table 1. This study included 18,034 participants, of which 52% were female and 48% were male. The prevalence of osteoarthritis in general stood at 9.8%. Individuals with elevated serum vitamin A levels exhibited a higher prevalence compared to those with lower levels [Q1(≤45.10 μg/dl): 6.4%, Q2 (45.10–55.40 μg/dl): 8.5%, Q3 (55.40–67.40 μg/dl): 10%, Q4 (≥67.40 μg/dl): 13%, *p* < 0.001]. Among participants with higher serum vitamin A, osteoarthritis prevalence was higher in Non-Hispanic White participants younger than 60.

**Table 1:** Baseline characteristics of participants with or without osteoarthritis according to serum vitamin A concentrations in NHANES 2001–2006 and NHANES 2017–2018 cycles.

			Vitamin A (μg/dl)				
Characteristic	*N*	Overall, *N* = 18,034 (100%)	Q1 (≤45.10), *N* = 4,514 (21%)	Q2 (45.10–55.40), *N* = 4,481 (25%)	Q3 (55.40–67.40), *N* = 4,527 (27%)	Q4 (≥67.40), *N* = 4,512 (27%)	*p* Value
Age, years	18,034						<0.001
<60		11,856 (76%)	3,528 (85%)	3,175 (81%)	2,835 (75%)	2,318 (67%)	
≥60		6,178 (24%)	986 (15%)	1,306 (19%)	1,692 (25%)	2,194 (33%)	
Sex	18,034						<0.001
Female		9,394 (52%)	3,169 (71%)	2,420 (55%)	1,992 (45%)	1,813 (41%)	
Male		8,640 (48%)	1,345 (29%)	2,061 (45%)	2,535 (55%)	2,699 (59%)	
Race	18,034						<0.001
Non-Hispanic White		8,608 (70%)	1,429 (53%)	1,936 (67%)	2,380 (74%)	2,863 (81%)	
Non-Hispanic Black		3,728 (11%)	1,301 (19%)	962 (11%)	777 (8.2%)	688 (6.5%)	
Mexican American		3,352 (7.9%)	1,090 (13%)	929 (8.9%)	793 (7.2%)	540 (4.0%)	
Other Race		1,435 (6.5%)	418 (8.4%)	397 (7.8%)	340 (5.6%)	280 (5.3%)	
Other Hispanic		911 (4.9%)	276 (6.6%)	257 (5.3%)	237 (5.0%)	141 (3.2%)	
Smoke (≥100 cigarettes in life)	18,034	8,462 (48%)	1,734 (41%)	2,066 (47%)	2,243 (49%)	2,419 (53%)	<0.001
BMI	18,034						<0.001
underweight		294 (1.7%)	117 (3.3%)	77 (1.8%)	50 (1.1%)	50 (1.2%)	
normal weight		5,090 (30%)	1,304 (32%)	1,270 (30%)	1,253 (28%)	1,263 (29%)	
overweight		6,282 (33%)	1,259 (25%)	1,513 (31%)	1,706 (37%)	1,804 (39%)	
obesity		6,368 (35%)	1,834 (40%)	1,621 (37%)	1,518 (34%)	1,395 (31%)	
Heart failure	18,034	582 (2.3%)	95 (1.6%)	105 (1.7%)	155 (2.5%)	227 (3.1%)	<0.001
Diabetes	18,034	2,070 (8.5%)	397 (7.3%)	442 (7.1%)	535 (8.4%)	696 (11%)	<0.001
Hypertension	18,034	6,026 (29%)	1,073 (21%)	1,282 (24%)	1,573 (29%)	2,098 (40%)	<0.001
Coronary heart disease	18,034	801 (3.7%)	98 (2.3%)	150 (2.9%)	214 (3.7%)	339 (5.6%)	<0.001
Stroke	18,034	663 (2.6%)	112 (2.0%)	152 (2.6%)	162 (2.4%)	237 (3.4%)	0.002
OA	18,034	1,682 (9.8%)	265 (6.4%)	337 (8.5%)	457 (10%)	623 (13%)	<0.001

Categorical variables were expressed using proportions (%) and analyzed using sampling weights.

### 3.2 Association of serum vitamin A with osteoarthritis

**Figure 2:**
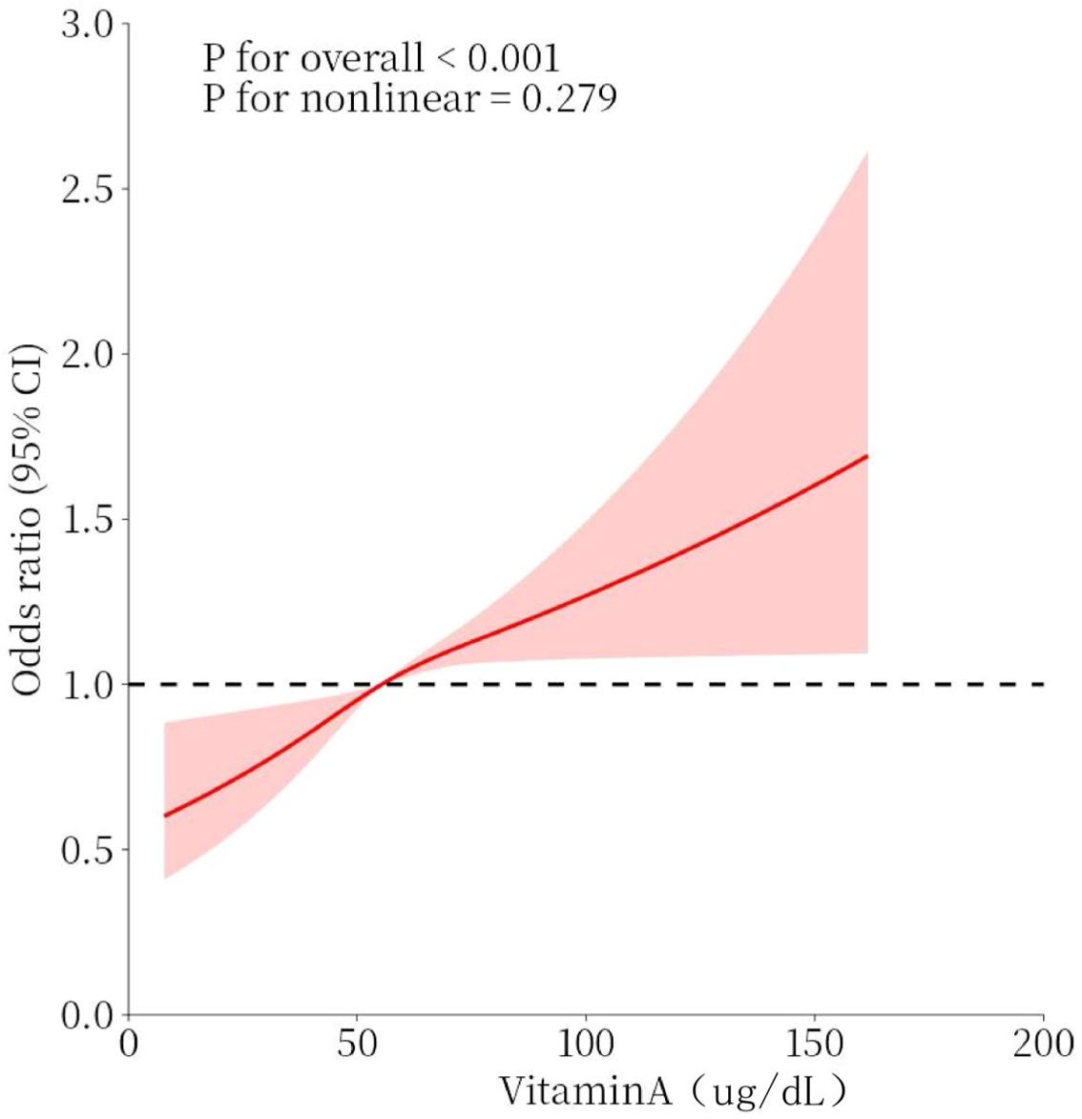
Dose–response relationship between serum vitamin A concentration and osteoarthritis. Models were adjusted for sex, age, race, BMI, smoking, diabetes, coronary heart disease, stroke, hypertension, and heart failure. The solid red line represents the OR for osteoarthritis and the red interval represents the 95% CI. overall *p*-value <0.001 demonstrated a significant association, and a non-linear association p-value >0.05 indicated a linear dose–response relationship.

The association between serum vitamin A (continuous variable) and osteoarthritis is shown in Figure 2, which adjusted for covariates and found that serum vitamin A was linearly associated with the risk of osteoarthritis, with the OR increasing as serum vitamin A increased. The results of this study suggest that vitamin A is a protective factor for osteoarthritis when Vitamin A < 55.4 μg/dl and a risk factor for osteoarthritis when Vitamin A ≥ 55.4 μg/dl.

**Figure 3:**
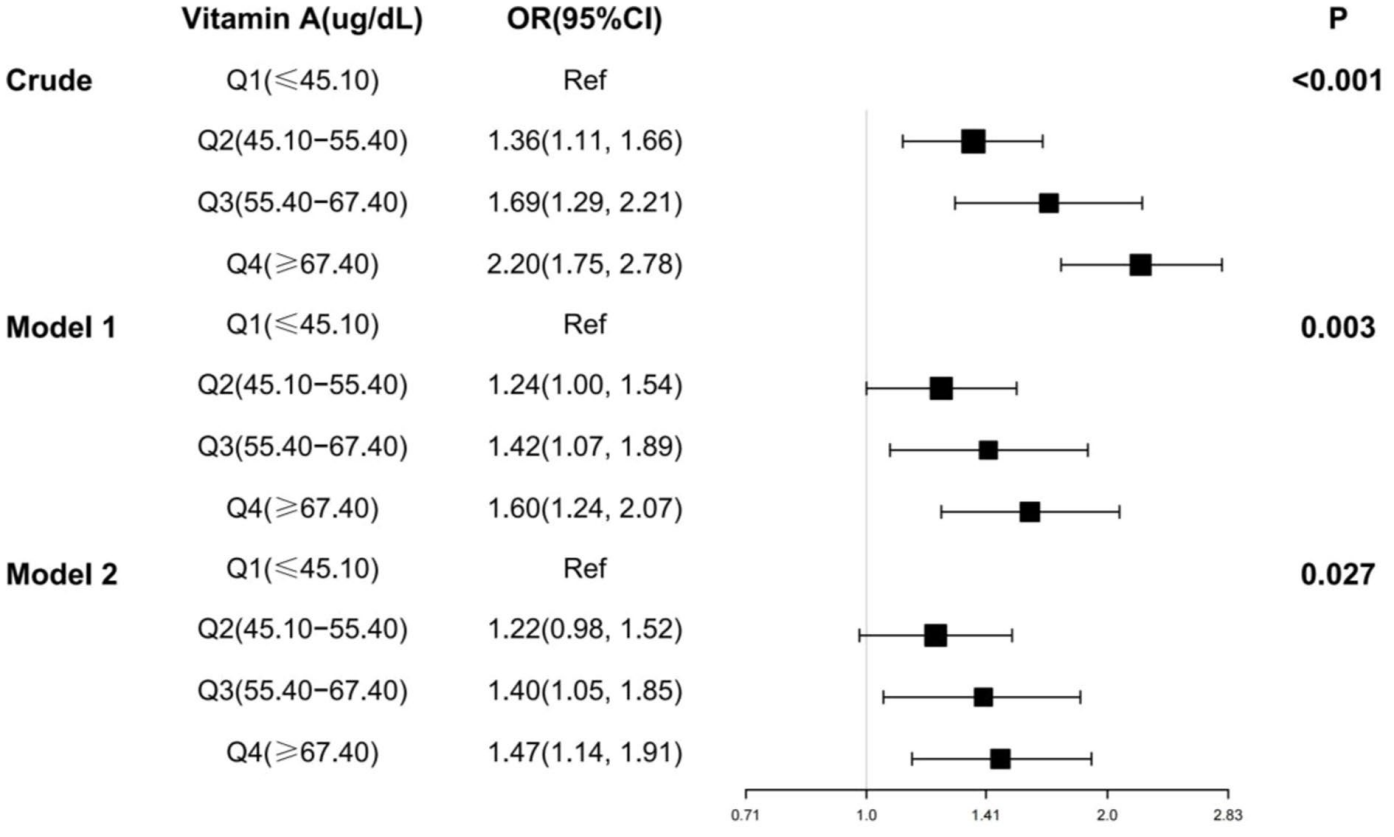
Multifactorial logistic regression analysis of serum vitamin A and osteoarthritis. OR, odds ratio; 95% CI, 95% confidence intervals. Crude: Unadjusted covariates. Model 1: Adjusted for gender, age and ethnicity. Model 2: Adjusted for gender, age, race, BMI, smoking, diabetes, coronary heart disease, stroke, hypertension, heart failure.

Multivariate logistic regression was also used in this study to analyze the relationship between serum vitamin A and osteoarthritis (Figure 3). The results showed that after adjusting for all covariates, the ORs for the four categories of serum vitamin A were 1.00 (reference), 1.22 (95% CI: 0.98,1.52), 1.40 (95% CI: 1.05, 1.85), and 1.47 (95% CI: 1.14,1.91), respectively, with a *p*-value of 0.027.

### 3.3 Stratification and sensitivity analysis

The results of the stratified analyses are shown in Table 2, stratified by age (≥60 and < 60), sex (Female and Male), race (Non-Hispanic White and Other), BMI (≥30 and <30), whether they had smoked more than 100 cigarettes in their lifetime (Yes and No), coronary heart disease (Yes and No), diabetes mellitus (Yes and No), hypertension (Yes and No), stroke (yes and no) and heart failure (yes and no) stratification. The results showed that the association between vitamin A and osteoarthritis was affected by sex, smoking status, diabetes, and stroke (p for interaction <0.05). At higher serum vitamin A, women who were nonsmokers and free of diabetes and stroke had a higher risk of developing osteoarthritis.

**Table 2:** Stratified analysis of serum vitamin A and osteoarthritis associations in participants in the NHANES 2001–2006 and NHANES 2017–2018 cycles.

			OR (95%CI)				
Characteristic	Count	Percent (%)	Q1 (≤45.10 μg/dl), *N* = 4,514	Q2 (45.10–55.40 μg/dl), *N* = 4,481	Q3 (55.40–67.40 μg/dl), *N* = 4,527	Q4 (≥67.40 μg/dl), *N* = 4,512	*p* for interaction
Age, years							0.073
<60	11,856	65.7	Reference	1.44 (1.11, 1.87)	1.72 (1.34, 2.22)	1.93 (1.49, 2.51)	
≥60	6,178	34.3	Reference	0.95 (0.76, 1.19)	1.18 (0.96, 1.46)	1.51 (1.24, 1.84)	
Sex							<0.001
Female	9,394	52.1	Reference	1.56 (1.28, 1.91)	2.61 (2.16, 3.16)	3.75 (3.12, 4.52)	
Male	8,640	47.9	Reference	1.05 (0.77, 1.42)	1.28 (0.96, 1.70)	1.98 (1.51, 2.60)	
Race							0.526
Non-Hispanic White	8,608	47.7	Reference	1.17 (0.93, 1.48)	1.62 (1.31, 2.01)	2.03 (1.66, 2.50)	
Other	9,426	52.3	Reference	1.21 (0.95, 1.55)	1.36 (1.06, 1.75)	2.04 (1.60, 2.61)	
Smoke (≥100 cigarettes in life)							0.001
No	9,572	53.1	Reference	1.60 (1.26, 2.02)	2.19 (1.74, 2.75)	3.36 (2.70, 4.18)	
Yes	8,462	46.9	Reference	1.01 (0.80, 1.28)	1.39 (1.12, 1.73)	1.84 (1.50, 2.27)	
BMI							0.694
<30	11,627	64.5	Reference	1.26 (0.99,1.59)	1.90 (1.53,2.36)	2.64 (2.14,3.24)	
≥30	6,407	35.5	Reference	1.42 (1.12,1.80)	1.81 (1.43,2.28)	2.79 (2.24,3.49)	
Heart failure							0.208
No	17,452	96.8	Reference	1.33 (1.12, 1.58)	1.81 (1.54, 2.13)	2.59 (2.22, 3.03)	
Yes	582	3.2	Reference	0.83 (0.40, 1.71)	1.16 (0.61, 2.20)	1.30 (0.72, 2.37)	
Diabetes							0.029
No	15,964	88.5	Reference	1.27 (1.05, 1.52)	1.87 (1.57, 2.22)	2.61 (2.21, 3.08)	
Yes	2070	11.5	Reference	1.40 (0.94, 2.08)	1.31 (0.89, 1.93)	1.83 (1.28, 2.61)	
Hypertension							0.207
No	12,008	66.6	Reference	1.20 (0.94,1.52)	1.63 (1.30,2.05)	2.28 (1.82,2.86)	
Yes	6,026	33.4	Reference	1.24 (0.98,1.57)	1.51 (1.21,1.89)	1.78 (1.44,2.20)	
Coronary heart disease							0.433
No	17,233	95.6	Reference	1.27 (1,07, 1.51)	1.69 (1.44, 1.99)	2.41 (2.06, 2.82)	
Yes	801	4.4	Reference	1.44 (0.72, 2.88)	2.23 (1.18, 4.24)	2.27 (1.23, 4.19)	
Stroke							0.003
No	17,371	96.3	Reference	1.31 (1.10, 1.56)	1.77 (1.50, 2.09)	2.64 (2.26, 3.09)	
Yes	663	3.7	Reference	0.96 (0.50, 1.85)	1.77 (0.97, 3.24)	1.15 (0.64, 2.07)	

Subgroup analyses adjusted for gender, age, race, BMI, smoking, diabetes, coronary heart disease, stroke, hypertension, and heart failure.

To further assess the robustness of the results, we performed sensitivity analyses excluding participants with serum vitamin A concentrations other than mean ± standard deviation × 3 (*n* = 192), those who were pregnant (*n* = 217), and those with a BMI ≥40 or BMI <15 (*n* = 1,121). Weighted multivariate logistic regression analyses were subsequently performed. The results in Table 3 showed that after harmonizing for confounders, the OR (95% CI) for the second, third, and fourth quartiles compared to the reference group in the lowest quartile were 1.24 (0.96,1.61), 1.47 (1.11, 1.95), and 1.51 (1.19,1.93), respectively. It can be seen that the positive correlation between serum vitamin A and the risk of osteoarthritis still exists. Subsequently, an unweighted multivariate logistic regression analysis was used and the results in Table 4 showed that after harmonizing the covariates, the OR (95% CI) for the second, third, and fourth quartiles compared to the reference group in the lowest quartile were 1.09 (0.91, 1.29),1.27 (1.07, 1.50), and 1.40 (1.19, 1.66), respectively. It can be seen that this relationship still exists, validating the association of a positive correlation between serum vitamin A and the risk of osteoarthritis, which becomes a risk factor for osteoarthritis when serum vitamin A > 55.4 μg/dl.

**Table 3:** Sensitivity analysis.

Group	Vitamin A (μg/dl)	OR	95% CI	*p*-value
Crude	Q1 (≤45.70)	Ref		<0.001
	Q2 (45.70–55.80)	1.41	1.10, 1.81	
	Q3 (55.80–67.50)	1.84	1.39, 2.42	
	Q4 (≥67.50)	2.30	1.82, 2.91	
Model 1	Q1 (≤45.70)	Ref		<0.001
	Q2 (45.70–55.80)	1.28	0.99, 1.66	
	Q3 (55.80–67.50)	1.52	1.14, 2.04	
	Q4 (≥67.50)	1.67	1.31, 2.14	
Model 2	Q1 (≤45.70)	Ref		0.005
	Q2 (45.70–55.80)	1.24	0.96, 1.61	
	Q3 (55.80–67.50)	1.47	1.11, 1.95	
	Q4 (≥67.50)	1.51	1.19, 1.93	

OR, odds ratio; 95% CI, 95% confidence intervals. Crude: Unadjusted covariates. Model 1: Adjusted for gender, age and ethnicity. Model 2: Adjusted for gender, age, race, BMI, smoking, diabetes, coronary heart disease, stroke, hypertension, heart failure.

**Table 4:** Unweighted multifactor logistic regression analysis.

Characteristic	Crude		Model 1		Model 2	
	OR (95%CI)	*p*	OR (95%CI)	*p*	OR (95%CI)	*p*
Vitamin A (μg/dl)						
Q1 (≤45.10)	Reference		Reference		Reference	
Q2 (45.10–55.40)	1.30 (1.10 ~ 1.54)	**0.002**	1.12 (0.95 ~ 1.34)	**0.181**	1.09 (0.91 ~ 1.29)	**0.352**
Q3 (55.40–67.40)	1.80 (1.54 ~ 2.11)	**<0.001**	1.34 (1.13 ~ 1.58)	**<0.001**	1.27 (1.07 ~ 1.50)	**0.005**
Q4 (≥67.40)	2.57 (2.21 ~ 2.98)	**<0.001**	1.58 (1.34 ~ 1.86)	**<0.001**	1.40 (1.19 ~ 1.66)	**<0.001**

OR, odds ratio; 95% CI, 95% confidence intervals. Crude: Unadjusted covariates. Model 1: Adjusted for gender, age and ethnicity. Model 2: Adjusted for gender, age, race, BMI, smoking, diabetes, coronary heart disease, stroke, hypertension, heart failure. Bold values in the table represent *p* -values.

## 4 Discussion

In the present study, we evaluated the association of serum vitamin A with osteoarthritis. This study included 18,034 participants from the NHANES 2001–2006 and NHANES 2017–2018 cohorts, and according to the results of the study, an elevated level of serum vitamin A was linked to an increased likelihood of developing osteoarthritis, with a linear correlation between the two. Subgroup analyses and sensitivity analyses also demonstrated this. In addition, gender, smoking, diabetes, and stroke may have an impact on the association between serum vitamin A and osteoarthritis.

Osteoarthritis is characterized by cartilage loss (19) involving the subchondral bone. Subchondral bone is at the distal end of calcified cartilage and plays an important role in maintaining joint flexibility and bone metabolism (20). Osteoclasts are multifunctional cells responsible for bone resorption and remodeling (21) and play a key role in subchondral bone loss in the early stages of osteoarthritis (22). They break down inorganic salts and organic substances present in bone tissue by adhering to the surface of the bone and releasing acids and enzymes from lysosomes to regulate the process of bone development and regeneration (23, 24). Osteoclasts are also closely linked to the immune system, secreting associated factors to promote inflammation (25). Osteoclasts are therefore critical to the pathogenesis of osteoarthritis. When osteoclasts are increased, the subchondral microenvironment is disrupted and bone resorption is markedly enhanced. This leads to increased loss of subchondral bone, decreased bone density, and enhanced joint irregularity, leading to joint discomfort and pain. Moreover, osteoclast-induced inflammation induces degradation of the cartilage extracellular matrix and promotes chondrocyte apoptosis (26), which further encourages the development of osteoarthritis. In patients with OA, the expression of retinoic acid receptor α coactivator protein is elevated (16), and the retinoid signaling pathway plays an important role in OA. *In vitro* studies have shown that vitamin A derivatives reduce type 2 collagen production and increase matrix metalloproteinase activity (17), one of the key enzymes in the progression of osteoarthritis (27), which in turn promotes OA formation. Khosasih et al. showed (28) that vitamin A promotes hand OA by regulating the downstream gene CYP26B1, which breaks down excess retinoic acid, the most active metabolic derivative of vitamin A. Increased expression of CYP26B1 decreases retinoic acid levels, and decreased bioavailability of retinoic acid damages cartilage (29) and aggravates Osteoarthritis. Retinol-binding protein 4 (RBP4), a member of the vitamin A family, is a carrier of serum vitamin A and is responsible for the transport of retinoic acid from the liver to peripheral tissues (30). Previous scholars have shown that RBP4 can interfere with insulin signaling by activating the PI3K/Akt pathway or the JAK2/STAT5 pathway (31, 32), and it is noteworthy that activation of PI3K/Akt or JAK signaling stimulates inflammation, cellular metabolism, and apoptosis in OA (33). RBP4 binds to Toll-like receptor 4 (TLR4). Upon interaction with TLR4, RBP4 activates c-Jun N-terminal kinases (JNK) and NF-κB. This activation further triggers the production of IL-1β and TNF-α, ultimately resulting in the impairment of insulin signaling and the development of insulin resistance (IR) (31). Notably, the development of IR is closely associated with the development of arthritis (34, 35), and TLR activation also leads to the production of pro-inflammatory cytokines in chondrocytes (36), which promotes OA. Most of the effects of vitamin A are mediated by its active metabolite all-trans retinoic acid (atRA), and Zhu et al.’s study (37) demonstrated that atRA metabolic blockers inhibit mechanical damage in OA, further confirming that vitamin A promotes OA.

We have found through our research that serum vitamin A is linearly correlated with the risk of osteoarthritis and that the prevalence of osteoarthritis progressively increases with increasing serum vitamin A concentrations. Vitamin A became a risk factor for osteoarthritis when serum vitamin A concentrations exceeded 55.4 μg/dl. This is consistent with the findings of previous studies on vitamin A and osteoarthritis. The association was more pronounced in women and was influenced by smoking status, diabetes status, and stroke status.

Vitamin A is an essential micronutrient in the human body (38), and it is known that vitamin A deficiency can lead to night blindness, dry eyes, tuberculosis, and anemia (39–41), and pregnant women and children are most likely to suffer from vitamin A deficiency. Therefore, it is essential for vitamin A supplementation, but too much serum vitamin A can cause bone damage. In the future, greater attention should be paid to the risk of vitamin A overdose, and additional clinical trials focusing on serum vitamin A levels are necessary to better understand the threshold effect of vitamin A.

## 5 Strengths and limitations

One of the main strengths of this study is the large sample size, using a nationally representative sample that adjusted for a large number of confounders such as gender, race, age, and comorbidities. Limitations of this study are that serum vitamin A is affected by a variety of conditions and that this study used a single measurement of serum vitamin A concentration, which may not be consistent with an individual’s long-term vitamin A status. However, we performed a series of sensitivity analyses, which still resulted in a positive correlation between serum vitamin A concentration and the risk of osteoarthritis. The diagnosis of osteoarthritis in this study was based on participants’ self-reports, “Has a doctor or other health professional ever told you that you had arthritis?” and “Which type of arthritis was it?,” without further confirmation of participants’ medical records. The lack of information on the severity and location of osteoarthritis may result in some early osteoarthritis being missed and have an impact on the results, but it is unlikely to affect them. Finally, given that the present study was cross-sectional and reverse causality could not be eliminated, clinical studies on serum vitamin A and osteoarthritis were then required in subsequent work.

## 6 Conclusion

In summary, this study is the first to examine the association between serum vitamin A and osteoarthritis using cross-sectional sections. We found a linear association between serum vitamin A and the risk of osteoarthritis, with higher serum vitamin A associated with a higher risk of osteoarthritis, and reported for the first time that this association was more pronounced in women. Smoking, diabetes, and stroke also affect this association. Vitamin A levels can be used as a potential early warning indicator to help identify people at high risk of OA, thus providing a basis for early intervention. It can also help to determine the distribution of vitamin A concentrations in different populations (e.g., different ages, genders, races, etc.), as well as the prevalence trend of OA, which can be used to provide a basis for the formulation of public health policies. The vitamin A concentration thresholds obtained in this study can help develop appropriate nutritional interventions and improve people’s dietary habits to prevent or slow down the development of osteoarthritis and reduce the burden on individuals and society. More refined experiments need to be designed to validate our findings in the future.

## Data Availability

Publicly available datasets were analyzed in this study. This data can be found at: https://www.cdc.gov/nchs/nhanes.
